# Thrombotic events following tocilizumab therapy in critically ill COVID-19 patients: a Façade for prognostic markers

**DOI:** 10.1186/s12959-020-00236-9

**Published:** 2020-09-09

**Authors:** Bassam Atallah, Wasim El Nekidy, Saad I. Mallah, Antoine Cherfan, Laila AbdelWareth, Jihad Mallat, Fadi Hamed

**Affiliations:** 1 Department of Pharmacy Services, Cleveland Clinic Abu Dhabi, Al Maryah Island, Abu Dhabi, United Arab Emirates; 2 Cleveland Clinic Lerner College of Medicine of Case Western Reserve University, Cleveland, OH USA; 3 School of Medicine, Royal College of Surgeons in Ireland–Bahrain, Al Sayh, Bahrain; 4 Department of Clinical Pathology, Cleveland Clinic Abu Dhabi, Al Maryah Island, Abu Dhabi, United Arab Emirates; 5 Critical Care Institute, Cleveland Clinic Abu Dhabi, Al Maryah Island, Abu Dhabi, United Arab Emirates; 6 Normandy University, UNICAEN ED 497, Caen, France

**Keywords:** COVID-19, Tocilizumab, Thrombosis, D-dimers, Prognostic markers

## Abstract

**Background:**

Hospitals in the Middle East Gulf region have experienced an influx of COVID-19 patients to their medical wards and intensive care units. The hypercoagulability of these patients has been widely reported on a global scale. However, many of the experimental treatments used to manage the various complications of COVID-19 have not been widely studied in this context. The effect of the current treatment protocols on patients’ diagnostic and prognostic biomarkers may thus impact the validity of the algorithms adopted.

**Case presentation:**

In this case series, we report four cases of venous thromboembolism and 1 case of arterial thrombotic event, in patients treated with standard or intensified prophylactic doses of unfractionated heparin or low molecular weight heparin at our institution. Tocilizumab has been utilized as an add-on therapy to the standard of care to treat patients with SARS-CoV-2 associated acute respiratory distress syndrome, in order to dampen the hyperinflammatory response. It is imperative to be aware that this drug may be masking the inflammatory markers (e.g. IL6, CRP, fibrinogen, and ferritin), without reducing the risk of thrombotic events in this population, creating instead a façade of an improved prognostic outcome. However, the D-dimer levels remained prognostically reliable in these cases, as they were not affected by the drug and continued to be at the highest level until event occurrence.

**Conclusions:**

In the setting of tocilizumab therapy, traditional prognostic markers of worsening infection and inflammation, and thus potential risk of acute thrombosis, should be weighed carefully as they may not be reliable for prognosis and may create a façade of an improved prognostic outcome instead. Additionally, the fact that thrombotic events continued to be observed despite decrease in inflammatory markers and the proactive anticoagulative approach adopted, raises more questions about the coagulative mechanisms at play in COVID-19, and the appropriate management strategy.

## Background

The COVID-19 (SARS CoV-2) pandemic has stricken all corners of the globe. Hospitals in the Middle East Gulf region, including the United Arab Emirates (UAE), have experienced an influx of coronavirus patients to their medical wards and intensive care units. With over 62,000 confirmed SARS-CoV-2 cases to date in the UAE [1], and a diverse population composed of locals and expatriates, it is expected that the various reported manifestations of the pandemic are to be observed in this country as well. Our quaternary care hospital has adapted to accommodate the influx by approximately doubling ICU bed capacity, and dedicating half of it for COVID-19 patients.

Coagulopathies of controversial mechanisms and etiologies have been widely reported in COVID-19 patients [2, 3]. Additionally, treatment approaches vary by institution, with many of the drugs being used in unique contexts and unchartered territory. As a result, devised algorithms for risk assessment, management, and prognosis may be based on markers atypically impacted by the adopted treatment regimens of novel COVID-19 patients.

## Case presentation

We report four cases of venous thromboembolism (VTE) and 1 case of arterial thrombotic event, in patients treated with standard or intensified prophylactic doses of unfractionated heparin or low molecular weight heparin at our institution. One patient experienced a second pulmonary embolism while on therapeutic intravenous heparin with an activated partial thromboplastin time (aPTT) goal of 60 to 85 s following a first VTE event. All patients were admitted to the ICU for SARS-CoV-2 infection. All five patients received tocilizumab prior to the thrombotic events.

Patient 1 is a 51-year-old male who received tocilizumab prior to transfer to our hospital. His D-dimer was over 6 times the upper limit of normal (ULN) and started on intensified prophylaxis with unfractionated heparin infusion (UFH) 7500 units TID per our COVID anticoagulation protocol [4]. His C-reactive protein (CRP) and fibrinogen were within normal limits, while ferritin and IL-6 were both elevated. On day 3 of admission, ischemia of the first 3 toes was noted with no capillary refill, and no pulse on bedside ultrasound (US). Patient anticoagulation plan was changed to heparin DVT/PE protocol (aPTT 60–85 s). CT angiogram showed partially detaching/floating thrombi noted 4 cm infrarenal abdominal aorta, compromising the lumen and leading to acute limb ischemia with extensive abdominal and bilateral lower extremity thrombus. D-dimer was still over 6 times ULN while all other markers were lower than at admission. Patient was screened for lupus anticoagulant (LAC), and returned negative. Patient developed secondary ischemia of bilateral lower extremity developing left foot dry gangrene requiring below-knee amputation. Patient eventually improved and was discharged on day 75 of admission on oral anticoagulation.

Patient 2, a 79-year-old male, showed no acute or chronic pulmonary embolism (PE) on CT chest with PE protocol at admission. CRP was elevated and D-dimer was over 6 times the upper limit of normal. He received DVT prophylaxis with Enoxaparin 40 mg subcutaneously (SC) daily. On Day 3 of admission, tocilizumab 400 mg intravenously was administered. Patient remained critically ill and on mechanical ventilation, having failed weaning trials. Two weeks into admission, he had a sudden increase in O2 requirement, which was contributed to by secondary bacterial infection. However, given prolonged ICU course and known COVID-19 status, DVT US of lower extremities was performed. DVT in bilateral calf veins was evident, and patient was started on full anticoagulation using unfractionated heparin infusion. His D-dimer was > 4 ULN at that time while CRP and ferritin were elevated, but much lower than admission. Almost 30 days post ICU admission, he suddenly went into Pulseless Electrical Activity rhythm. Bedside focused ICU ECHO showed very subtle cardiac motion, no pericardial effusion, no carotid or femoral arteries pulsation, and with evidence of large clot in the right atrium, likely representing massive PE. As patient was already on comfort care measures, tissue plasminogen activator was not given and patient was pronounced dead. Patient was not screened for lupus anticoagulant.

Patient 3 is a 31-year-old male, with thalassemia trait and mild intermittent asthma, admitted with flulike symptoms. Patient was hypoxemic on presentation, and was transferred to the ICU with SPO2 in the 80s requiring nonrebreather oxygen mask. CXR showed pulmonary infiltrates. COVID-19 RT-PCR test was positive from day 1, and patient received tocilizumab on same day. Biomarkers on admission showed a D-dimer elevation of 3 times ULN. Patient was placed on standard dose DVT prophylaxis with low molecular weight heparin. A point of care US performed on day 7 in response to bilateral lower limb pain and D-dimer elevation to over 6 times ULN revealed occlusive deep vein thrombosis of the right distal femoral, popliteal, posterior tibial, and peroneal veins, as well as the left popliteal, gastrocnemius, and peroneal veins. Patient thromboprophylaxis was escalated to therapeutic dose anticoagulation and was then transferred to a medical floor 2 days later. On day 10 of admission, a CTPA performed in response to worsening pleuritic chest pain revealed subsegmental pulmonary embolus within the right lower lobe basilar pulmonary artery. Treatment strategy was thus switched to heparin therapeutic intravenous drip. Patient continued to improve and was subsequently discharged on oral anticoagulation with apixaban on day 17 of admission. Patient was screened for LAC and returned positive.

Patient 4 is a 36-year-old male with unremarkable past medical history, presented with fever and chills ongoing for 8 days. COVID-19 RT-PCR test was positive from day 1 of admission. CXR showed left mid-lung mild airspace opacities, with patient initially treated on a medical floor. Admission D-dimer was only slightly elevated, and patient was placed on standard DVT prophylaxis. On day 3 of admission patient was transferred to the ICU for worsening hypoxia and received tocilizumab. D-dimer continued to progressively increase and reached 6 times ULN on day 7; patient was thus placed on high intensity prophylaxis with enoxaparin 40 mg BID. On day 11 of admission a CTPA obtained due to progressive hypoxia revealed large saddle embolus. Extension of embolus into the upper and lower lobar pulmonary arteries was evident, with resulting right heart strain. Patient received systemic alteplase followed by heparin drip for therapeutic anticoagulation. Patient improved and was transferred to a medical ward on day 14 of admission and eventually discharged home on oral anticoagulation on day 21 of admission. Patient was screened for LAC and returned positive.

Patient 5, a 51-year-old male, with history of hypertension transferred from outside hospital to the ICU at our institution on mechanical ventilation following severe COVID-19 ARDS. D-dimer was over 6 times ULN on transfer: Patient was directly placed on high intensity thromboprophylaxis with heparin 7500 SC TID and received tocilizumab on second day of admission. Patient’s overall condition continued to worsen, developing acute kidney injury requiring continuous renal replacement therapy. CT chest with IV contrast obtained on day 11 to rule out collection as source of infection revealed bilateral PE, and patient was thus commenced on therapeutic IV heparin. D-dimer continued to be 6 times ULN throughout admission and up until day of event. Patient was screened for LAC and returned positive. His condition continued to deteriorate, developing bacteremia and cavitary lung disease with confirmed aspergillosis. Patient was treated with anti-fungal but progressed to invasive form with hemoptysis. Patient had a cardiac arrest and expired on day 76 of admission. All data related to the five patients is outlined in Table 1.

**Table 1:** Patient baseline characteristics and parameters for inflammation and coagulopathy.

Variable	Patient 1	Patient 2	Patient 3	Patient 4	Patient 5
**Age (yrs)**	51	79	31	36	51
**BMI (kg/m**^**2**^**)**	26	33	30	30.7	22.6
**Sex**	Male	Male	Male	Male	Male
**Past Medical History**	Diabetes	Prostate Carcinoma (remission)	Thalassemia, Asthma	None	Hypertension
**Admission Thromboprophylaxis Regimen**	Heparin SC 5000 TID - Heparin SC 7500 TID on Day 2, based on D Dimer over 3	Heparin SC 5000 TID	Enoxaparin 40 mg daily	Enoxaparin 40 mg daily, increased to 40 mg BID on day 7	Heparin 7500 TID
**Signs of thrombosis**	Day 3: On observation, ischemia of the first 3 toes, no capillary refill	Day 17: No specific signs, bilateral lower extremity ultrasound ordered based on persistently high D Dimer	Day 7: Bilateral lower limb pain	Day 11: No specific signs but CT angiogram obtained for persistent severe hypoxemia and high D Dimer	Day 13: No specific signs but CT chest with IV contrast obtained to rule out collection
**Imaging Requested**	CT Angiogram (abdomen and pelvis with lower extremity runoff): Floating thrombi noted 4 cm infrarenal abdominal aorta compromising the lumen with total obliteration of the right popliteal artery and right anterior tibial artery	Bilateral lower extremity Ultrasound: The right posterior tibial vein appears dilated with visible thrombus	Bilateral lower extremity Ultrasound: Occlusive deep vein thrombosis of the right distal femoral, popliteal, posterior tibial, and peroneal veins, and the left popliteal, gastrocnemius and peroneal veins	CT Angiogram Chest: Large saddle embolus, extension of embolus into the upper and lower lobar pulmonary arteries with right heart strain	CT Chest with IV Contrast: Multiple filling defects noted bilaterally in the pulmonary tree; findings consistent with pulmonary embolism. Small filling defect seen in the internal jugular veins bilaterally, consistent with thrombi.
**Vascular Intervention**	1.Percutaneous mechanical thrombectomy of the infrarenal aorta 2. Left lower extremity intra-arterial thrombolysis	None	None	None	None
**Pharmacologic Antithrombotic Therapy for Confirmed thrombosis**	Heparin Drip per DVT/PE protocol (aPTT target 60–85)	Enoxaparin 1 mg/kg BID from day 17 till day 29 of admission when patient developed AKI and switched to heparin drip	Enoxaparin 1 mg/kg BID, switched to heparin drip on day 10 of admission	Systemic alteplase followed by heparin DVT/PE protocol (aPTT target 60–85)	Heparin Drip per DVT/PE protocol (aPTT target 60–85)
	Secondary ischemia of bilateral lower extremity, developing left foot dry gangrene requiring below-knee amputation. Patient improved and discharged on oral anticoagulation.	Expired on day 38 of admission, while on heparin drip therapeutic dose for DVT/PE (aPTT target 60–85), CRRT, and mechanical ventilation. Potential PE	Discharged on oral anticoagulation with apixaban on day 17 of admission	Patient improved and was transferred to a medical ward on day 14 of admission and eventually discharged home on oral anticoagulation on day 21 of admission	Developed bacteremia and cavitary lung disease with confirmed aspergillosis, which progressed to invasive form with hemoptysis. Patient had a cardiac arrest and expired on day 76 of admission while on therapeutic IV heparin and mechanical ventilation.
**Tocilizumab**	Prior to admission at OSH	Day 3	Day 1	Day 3	Day 2
**D-Dimer (mcg/mL)**					
**Admission**	Over 4	Over 4	2.05	0.54	Over 4
**At time of event**	Over 4	Over 4	Over 4	Over 4	Over 4
Platelets (×10*9/L)					
**Admission**	311	180	311	201	278
**At time of event**	218	161	218	474	515
**C-Reactive protein (mg/L)**					
**Admission**	8	193	63.2	59.3	307.5
**At time of event**	3.8	49	3.8	3.5	297.2
**Fibrinogen (g/L)**					
**Admission**	1.88	NA	6.57	4.53	7.06
**At time of event**	1.4	5.01	3.29	2.53	NA
**Lupus Anticoagulant**	Negative	N/A	Positive	Positive	Positive

## Discussion and conclusions

COVID-19 has been demonstrated to be widely implicated from a hematologic point of view. Pooled results from a meta-analysis of nine studies (*n* = 1105 patients) describing COVID-19 patient characteristics revealed that prothrombin time (PT) and D-dimer levels were significantly higher in patients with severe COVID-19 (0.68, 95% CI = 0.43–0.93, *I* ^2^ = 53.7%, 0.53, 95% CI = 0.22–0.84, *I* ^2^ = 78.9%, respectively) [4]. A similar meta-analysis that included 22 Chinese studies (*n* = 4889 confirmed COVID-19 inpatients) found that severe patients had significantly higher D-dimer levels and prolonged PT compared with non-severe patients. Furthermore, non-survivors had significantly higher D-dimer levels, prolonged PT, and decreased platelet count compared with survivors [5]. Likewise, such findings have been observed and reported in different ethnicities and countries worldwide [2, 3].

Virus-mediated impacts on the immune system and its respective response, such as systemic inflammation and complement-system activation (cytokine storm), have been hypothesized to affect coagulation and hemostasis. Specific immune responses, such as an increase in white blood cells, may interact with and activate platelets, resulting in a clotting domino effect, and may possibly directly contribute to coagulopathy by forming platelet-leukocyte aggregates. Moreover, considering the vital role played by the endothelium in maintaining hemostasis and inducing coagulation, and SARS-CoV-2’s cell entry via ACE2 receptors (which are prevalent in endothelial cells), the virus may be directly implicated in the thrombotic events witnessed as a result of endothelial injury and inflammation (endothelialitis) [6]. In fact, recent evidence implicated COVID-19 as a vascular disease [7], with a new lung autopsy report showing unique features of severe endothelial injury associated with the presence of intracellular virus and disrupted cell membranes, as well widespread thrombosis with new vessel growth in COVID-19 patients [8]. Finally, the fact that most patients who were screened for LAC came back positive, is in-line with previous reports on the prevalence of LAC in COVID-19 patients [9]; a possible mechanism behind the increased clotting tendency. A recent prospective observational study confirms the frequent presence of single LAC positivity during acute phase of COVID-19 infection, but argues against a clear relation to thrombotic events. The study also notes that triple antiphospholipid positivity was very rare [10]. Nonetheless, many of these factors may potentially be involved in the observed increase of coagulation, particularly in severe infections. Therefore, markers of inflammation have been integrated in the overall suggested algorithms to both assess disease severity, and, potentially, the pending risk of clot formation.

**Fig. 1:**
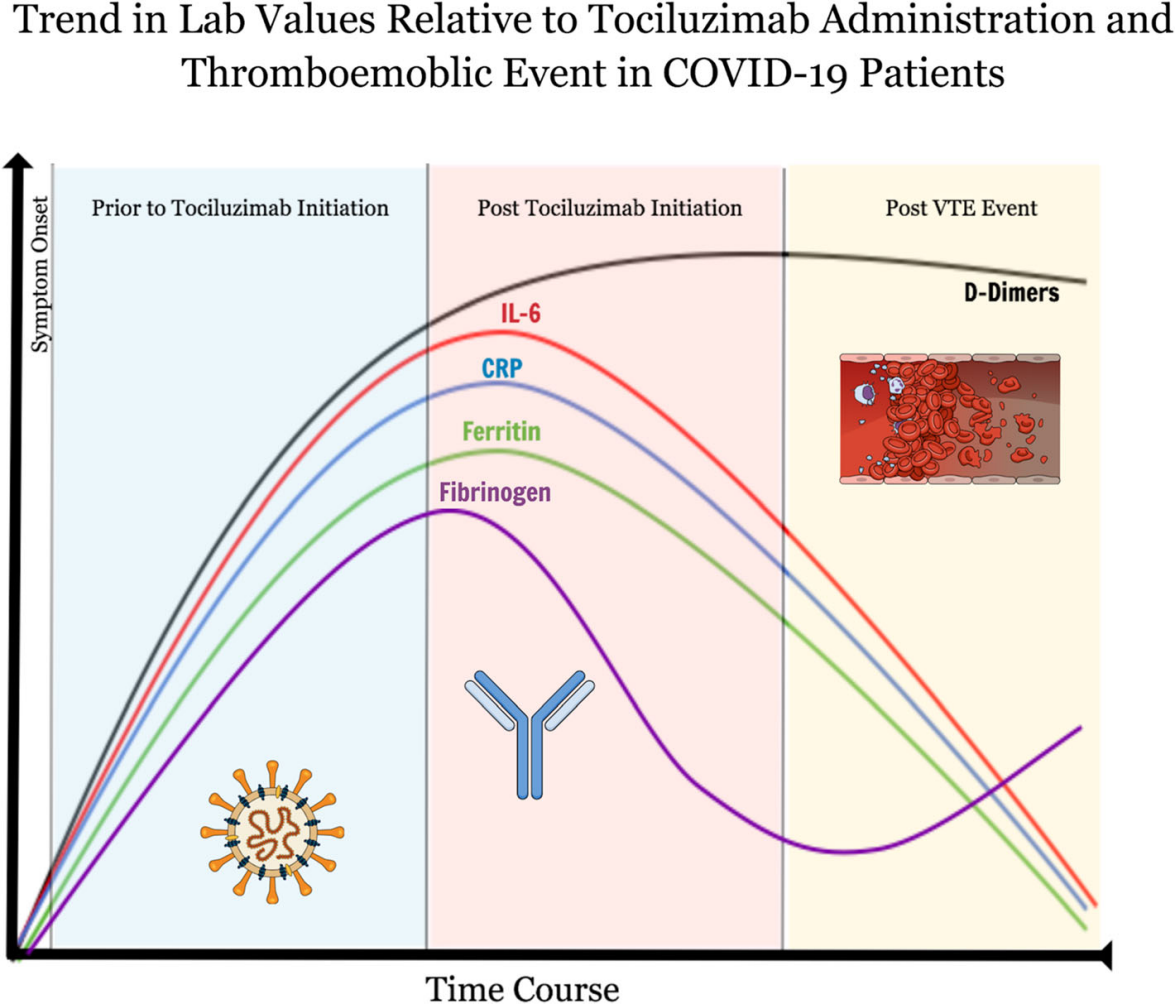
General trend observed in lab values of COVID-19 patients prior to and post tocilizumab therapy and thrombotic events (not based on exact patient values)

At our institution, as in many others [11], we have been utilizing tocilizumab, a recombinant humanized anti-IL6 monoclonal antibody produced in mammalian cells [12], as add-on therapy to the standard of care to treat patients with SARS-CoV-2 associated ARDS. The purpose is to dampen the hyperinflammatory response, and thus control the associated SARS-CoV-2 cytokines storm. All five patients that we report here received tocilizumab prior to the first thrombotic event. It is thus imperative to be aware that this drug could potentially mask the inflammatory markers (e.g. IL6, CRP, fibrinogen, and ferritin) [13–15], without reducing the risk of thrombotic events in this population, creating instead a façade of an improved prognostic outcome. The reduction however is fairly heterogenous in our small sample size, making larger studies necessary in order to map out and better understand the trend. In fact, a retrospective study in this context have demonstrated variable influences of tocilizumab on markers such as IL-6 and CRP [16]. Other retrospective cohort analyses however report results similar to our findings, with majority of patients experiencing an elevation of D-dimers after Tocilizumab administration, while the inflammatory markers (ferritin, LDH and CRP) gradually decreased [17, 18]. Regardless, what can be asserted with more certainty is that the D-dimer levels remained prognostically reliable in these cases, as they were not affected by the drug and continued to be at the highest level until event occurrence (Fig. 1). Additionally, the fact that thrombotic events continued to be observed despite decrease in inflammatory markers and the proactive anticoagulative approach adopted, raises more questions about the coagulative mechanisms at play in COVID-19, and the appropriate management strategy. These findings remain observational nonetheless and open to bias and limitations. Randomized controlled trials will be necessary before any major change in policy.

In view of the increased thrombotic events documented globally [2–5], including at our institution, it is important to be aware of the effects of the current suggested COVID-19 regimens, such as tocilizumab, on biomarkers that may be integrated into the anticoagulation algorithms. Importantly, in the setting of tocilizumab therapy, traditional prognostic markers of worsening infection and inflammation, and thus potential risk of acute thrombosis, should be weighed carefully as they may not be reliable for prognosis.

## Data Availability

Made available in Table 1, and via correspondence with BA.
